# 

**Published:** 1878-07

**Authors:** 


### PENNSYLVANIA STATE DENTAL SOCIETY.

The executive committee take pleasure in announcing the following programme for the meeting of this Society at Bedford Springs, Penn., commencing July 30th, at ten o'clock a. m., and continuing three days,

ESSAYS.

1. Thirty Years' Experience with Anæsthetics. By S. Welchens, D. D. S.
2. A Review of the Conservative Treatment of the Dental Pulp. By Louis Jack, D. D. S.
3. Anti and Post Professional Education. By H. Gerhart, D. D. S.
4. The Accepted Creed. By Prof. C. N. Pierce, D. D. S.
5. Gold and Tin in Combination. By Prof. D. D. Smith, D. D. S.
6. "Artificial Crowns." By Prof. E. T. Darby, D. D. S.

7. Why do Fillings Fail? The Electro-chemical Theory of the "New Departure" *vs.* Defective Manipulation and Defective Structure. By W. H. Trueman, D. D. S.

8. "Tissues." By Marshall H. Webb, D. D. S.

9. Discoloration of the Teeth. By G. W. Adams, D. D. S.

10. Subject to be announced. By R. Huey, D. D. S.

11. Address of the retiring President. By C. S. Beck, D. D. S.

SURGICAL CLINICS.

A tumor, the inferior maxillæ extending from the first molar on the left side to the first bicuspid on the right side will be removed. By Prof. Chas. J. Essig, D. D. S.

OPERATIVE CLINICS.

Cohesive Gold impacted against and over frail enamel, so that such walls may be encased and protected by gold and restoration of the contour of missing tissue by the aid of a new Electro-Magnetic Mallet. By Marshall H. Webb, D. D. S.

The use of Williams' Cylinders illustrated. By Rob't Huey, D. D. S.

Restoration of contour by the aid of the Electro-Magnetic Mallet. By Geo. B. McDonnald, D. D. S.

Operation with the Electro-Magnetic Mallet. By E. P. Kremer, D. D. S.

OPERATION.

By H. C. Longnecker, D. D. S.

A number of new appliances will be exhibited by inventors, including file carriers, syringes, chairs, engines and many others by manufacturers.

The committee desire to make this an interesting feature of the meeting, and will make suitable provision for the exhibition of any inventions or objects of interest to the society that may be presented.

G. W. KLUMP,
Chairman Executive Committee,
Williamsport, Pa.

### Editorial.

### ALUMNI REUNION.

At the close of the recent session of the Ohio College of Dental Surgery, it was decided by those interested who were present to hold a reunion of all the alumni at the time of the next annual commencement probably on the first Tuesday of March, 1879; when it is proposed to have every class that has ever gone out from the institution as fully represented as possible. We hope that very living member will be present at that time.

A general committee consisting of one member of each class was appointed, whose duty it shall be to interest their respective classes in the meeting, and to do whatever they may for the interest and success of the occasion. That committee is as follows, viz:

A. Berry, Cincinnati, O., of class 1846; A. S. Talbert, Lexington, Ky., 1847; J. S. King, Pittsburg, Pa., 1848; E. Bray, Evansville, Ind., 1849; J. Ramsey, Springfield, O., 1850; J. C. Clark, Circleville, O., 1851; J. C. Winnery, Salem, O., 1852; Geo. W. Keely, Oxford, O., 1853; Geo. Watt, Xenia, O., 1854; R. C. Reid, Anderson, Ind., 1855; E. Osmond, Cincinnati, O., 1856; H. A. Smith, Cincinnati, O., 1857; S. L. Bracy, Raymond, Miss., 1858; E. Ruth, Maysville, Ky., 1859; J. A. Watling, Ypsilanti, Mich., 1860; Geo. S. Allen, New York, 1861; Wm. Taft, Cincinnati, O., 1863; W. N. Morrison, St. Louis, Mo., 1864; J. I. Taylor, Cincinnati, O., 1865; C. R. Taft, Cincinnati, O., 1866; Henri L. Ambler, Cleveland, O., 1867; J. P. Holmes, Terry, Miss., 1868; J. A. Cassidy, Covington, Ky., 1869; J. M. Porter, Toledo, O., 1870; J. E. Cravens, Indianapolis, 1871; J. H. Warner, Columbus, O., 1872; T. S. Hacker, Indianapolis, Ind., 1873; L. L. Dunbar, San Francisco, Cal., 1874; C. B. Mower, Worster, O., 1875; C. I. Keely, Oxford, O., 1876; A. L. Brown, Cincinnati, O., 1877.

An executive committee was appointed consisting of J. Taft, H. A. Smith, and E. G. Betty, of Cincinnati, and J, S.

Cassidy, of Covington, Ky., whose duty it shall be to make the requisite arrangements.

Though we are not now authorized to specify the details, it may not be amiss to suggest, that on Thursday, March 6th, at two o'clock, p. m., the meeting be organized, and the afternoon devoted to a historical address and biographical sketches of those who are not living.

After this, if time permits in the afternoon, a full report from each class in the order of graduation, these reports to embrace all matters of special interest pertaining to each class, (this part of the programme to be finished in the evening if not in the afternoon). The evening to be devoted to an entertainment, sentiments, toasts, music, etc.

As one of the alumni of this institution we look forward to this reunion with very great interest.

The coming thus together of the children one common household or family, and renewing acquaintance, looking into each other's faces, and recounting the trials and struggles of our student days and college life, will be pleasant indeed, and make us one and all more fully appreciate our beloved alma mater, and stimulate us, pledge ourselves anew to give her that support and aid, which possibly some of us have sometimes been inclined to neglect.

Then let us come together and strengthen the bands of professional brotherhood by bringing into play the memories and associations of early days and first acquaintance.

### HO, TO AMERICAN DENTAL ASSOCIATION.

Arrangements have been made with the A. & G. W. R. R., to take persons to the American Dental Association at Niagara, at a special rate that will be acceptable to all desiring to go. Any who desire to go by this route and under this arrangement will please notify me as early as convenient, and I will procure tickets for them.

J. TAFT.

### CONTENTS.

COMMUNICATIONS.

Oral Electricity. *By J. S. Cassidy, D. D. S.,* ........................ 265

Pivot Teeth. *By E. Peabody,* ........................ 271

Professional Duties of the Dentist. *By C. L. Kelsey, D. D. S.,* ........ 278

Transplanting. *By G. R. Thomas, D. D. S.,* ........................ 281

PROCEEDINGS OF SOCIETIES.

Discussions before the Kentucky State Dental Society ........................ 298

Pennsylvania State Dental Society ........................ 305

EDITORIAL.

Alumni Reunion ........................ 307

Resignation and Reorganization ........................ 309

Obituary ........................ 310

Ho, To American Dental Association ........................ 310

---

### *THE DENTAL REGISTER,*

**A MONTHLY MAGAZINE DEVOTED TO THE INTERESTS OF**

### THE DENTAL PROFESSION.

***Terms Reduced to $2 50 per Year. Single Copies 25c.***

### *EDITED BY PROF. J. TAFT, D. D. S.,*

AND PUBLISHED BY

SPENCER & CROCKER, Ohio Dental Depot.

The volume commences in January and closes in December.

The REGISTER being issued monthly is always fresh, therefore Indispensable to the practitioner who desires to keep posted up with the new inventions and improvements which are daily developed. Neither expense nor effort will be spared to give value and variety to its contents. Articles will be illustrated with well executed engravings whenever they will add to the interest or assist to explanation.

Its extensive circulation and frequency of issue make it one of the BEST DENTAL ADVERTISING MEDIUMS in the United States.

All subscrptions must begin with January or July.

Local or special notices, five lines of less, will be inserted for $3.00 each insertion; over five lines, 50 cts. per line.

All advertising bills payable in advance, or at furthest, after the issue of the first number containing the advertisement. All transient advertisements to be paid for in advance.

☞CONTRIBUTIONS SOLICITED.

Exclusively Editorial Communications should be addressed to Editor.

The latest number of the REGISTER may be ordered with or without other goods at any time, and all letters should be addressed to

**SPENCER & CROCKER, Ohio Dental Depot, Cincinnati, O.**

